# Thrombus Composition in Cerebral Venous Thrombosis

**DOI:** 10.1155/srat/8650226

**Published:** 2025-06-23

**Authors:** Ghil Schwarz, Angelo Cascio Rizzo, Martina Di Como, Amedeo Cervo, Antonio Macera, Guglielmo Carlo Pero, Maria Costanza Aquilano, Beatrice dell'Acqua, Marco Bacigaluppi, Francesco Ruggieri, Arturo Chieregato, Emanuela Bonoldi, Mariangela Piano, Maria Sessa, Elio Clemente Agostoni

**Affiliations:** ^1^Department of Neurology and Stroke Unit, ASST Grande Ospedale Metropolitano Niguarda, Milan, Italy; ^2^Department of Hematology, Oncology and Molecular Medicine, ASST Grande Ospedale Metropolitano Niguarda, Milan, Italy; ^3^Department of Neuroradiology, ASST Grande Ospedale Metropolitano Niguarda, Milan, Italy; ^4^Neuroimmunology Unit and Neurology Department, Institute of Experimental Neurology, IRCCS, San Raffaele Hospital, Milan, Italy; ^5^Department of Neurointensive Care Unit, ASST Grande Ospedale Metropolitano Niguarda, Milan, Italy

## Abstract

**Background and Aims:** Histological analysis of thrombi can enhance the understanding of pathophysiology. We aimed to analyze EVT-retrieved thrombi in cerebral venous thrombosis (CVT), compare them with acute ischemic stroke (AIS) thrombi, and correlate their composition with CT density.

**Methods:** Retrospective case-series, including five CVT and 10 AIS cases treated with EVT. Thrombus sections were stained with hematoxylin and eosin; Picro Mallory for RBCs, fibrin, and collagen; and Prussian Blue for iron plus immunohistochemical staining with anti-CD61 (platelets), anti-MPO (neutrophils), anti-CD3 (T-cells), anti-CD20 (B-cells), anti-CD34 (endothelial cells), anti-CD68 (macrophages), and anti-citH3 (NETs). Thrombus components were quantified (Orbit) and expressed as a percentage of total area. The CVT-thrombus relative density (rHU) was calculated as HU thrombus/HU contralateral.

**Results:** All CVT cases showed extensive thrombosis. Four patients had prior anticoagulation, and four had rHU > 1.00 with CT hyperdensity. The etiologies were heterogeneous. CVT thrombi were rich in red blood cells and displayed variable histological features, including signs of early organization. Compared to arterial thrombi, venous thrombi exhibited larger size (surface area 185.6 mm^2^ [IQR 83.0–237.9] vs. 21.8 mm^2^ [IQR 8.8–77.8]; *p* = 0.028) and lower fibrin content (16.6% [IQR 13.9–31.5] vs. 46.5% [IQR 25.1–49.5]; *p* = 0.036), with no other significant differences in composition. Low fibrin content and high RBC-to-fibrin ratio (*R* −0.9 and R 0.9, respectively; *p* = 0.047 for both) showed a significant correlation with rHU.

**Conclusion:** Our exploratory study first shows that CVT thrombi are larger than AIS thrombi, with higher RBC content and lower fibrin, matching CT density. These findings enhance the understanding of CVT pathophysiology but need validation.

## 1. Introduction

Cerebral venous thrombosis (CVT) represents a significant cause of stroke among young adults [1], emphasizing the need for timely diagnosis and treatment to prevent potential mortality or long-term disability [2]. Guidelines regarding acute medical treatment of CVT have not been changed since 2011 and recommend initial anticoagulation [3]. Despite a recent randomized controlled trial (RCT) [4] questioning the efficacy of endovascular thrombectomy (EVT) in CVT management, EVT may still be considered a treatment option for patients with inadequate response to anticoagulant therapy, early clinical deterioration, or extensive CVT burdens [5].

Histological evaluation of thrombus has gained importance in acute ischemic stroke (AIS) cases, aiding in the identification of stroke etiology and potentially influencing treatment strategies [6]. Indeed, thrombus composition, which also varies depending on the underlying etiology and influences treatment response [6], has emerged as a crucial factor in imaging characteristics and thrombus mechanical properties. The presence of red blood cell (RBC)–rich thrombi is associated with higher recanalization rates [7]. Conversely, the fibrin content impacts the risk of thrombus fragmentation [8]. Moreover, the known correlation between thrombus composition and CT density [7] can assist in estimating its composition and mechanical behavior during the acute phase, based on initial noncontrast CT (NCCT), potentially influencing the choice of device for treatment.

To date, histological data on thrombi retrieved from CVT patients undergoing EVT are extremely limited, with only a single case report available describing a recent thrombus predominantly composed of RBCs [9]. Nevertheless, similar to ischemic stroke, thrombus assessment in CVT could provide valuable insights into the disease's pathophysiology, guiding treatment strategies, fostering advancements, and supporting the development of dedicated endovascular devices for venous sinuses.

The aim of this study is to describe the histological composition of ex vivo thrombi obtained from patients who underwent EVT for CVT, compare them with thrombi from AIS patients, and evaluate whether the correlation between thrombus composition and CT density, as observed in arterial thrombi, is also confirmed in CVT thrombi.

## 2. Methods

### 2.1. Study Population

We conducted a retrospective analysis of patients with CVT who underwent EVT at Grande Ospedale Metropolitano Niguarda and San Raffaele Hospitals in Milan, Italy, between 2020 and 2023. Decisions regarding EVT were made on an individual basis by a multidisciplinary team consisting of neurologists and interventional neuroradiologists, according to the most recent international guidelines [3–10]. For comparison, we randomly selected an equivalent number of EVT-treated AIS cases attributed to large-artery atherosclerosis (LAA) and an equivalent number due to cardioembolic (CE) etiology from the stroke unit database of Niguarda Hospital within the same time frame. Ischemic stroke etiology and CVT etiology were classified after extensive diagnostic assessment, with the former classified per the TOAST (trial of ORG 10172 in acute stroke treatment) classification [11]. For each patient, we retrieved baseline clinical characteristics, anticoagulant therapy before EVT, onset-to-EVT time, severity of presenting symptoms (baseline NIHSS score), vascular occlusion site on baseline CT-angiography, EVT-related details (including type of anesthesia, device/technique, number of passes, modified treatment in cerebral infarction (mTICI) scale [12] for AIS, EVT-related complications [including vessel dissection and perforation]), hemorrhagic transformation (defined per the ECASS (European Cooperative Acute Stroke Study)-II definition [13]) and 90-day functional outcome (via Modified Rankin Scale [14]).

### 2.2. Thrombus Density on NCCT Scan

CT density measurements were performed on the NCCT scan acquired prior to EVT, which may not have been the initial imaging study in CVT cases. The area of density variation was initially identified through a general visual assessment (categorized as present vs. absent) by single neurologist on NCCT scans—if the density of the lumen of any intracranial artery (in AIS cases) or venous sinus (in CVT cases) appeared higher/lower than the density of the adjacent or corresponding artery or venous sinus on the contralateral side. In this area, three adjacent circular regions of interest (ROIs) were drawn (each measuring around 0.02 cm^2^). The density in Hounsfield units (HUs) measured in the three ROIs was then averaged ([ROI1 + ROI2 + ROI3]/3) to obtain the absolute density of the thrombus. By using three analogous ROIs in the mirror area, the thrombus density was adjusted to obtain the relative density of the thrombus (rHU = HU *T*hrombus/HU *C*ontralateral). In cases of venous thrombosis in an odd and median sinus, lacking a mirror counterpart, the adjustment ROIs were obtained from the transverse sinuses. To assess intrarater reliability, each NCCT scan was independently evaluated twice by the same neurologist, with a 2-month interval between assessments. Inter-rater reliability was assessed by comparing the neurologist's ratings with those of an experienced neuroradiologist.

### 2.3. Thrombus Assessment

Thrombotic material collected during EVT was immediately fixed in 10% formalin solution, neutral buffered, embedded in paraffin, and cut in serial sections of 2 *μ*m. Thrombus sections were stained with hematoxylin and eosin (H&E), Picro Mallory Kit for Special Stain (DIAPATH), and Iron Staining Kit Ventana (Prussian blue stain) with BenchMark Special Stain (Roche-Ventana). H&E staining was employed for morphological description; Picro Mallory was used to quantify RBCs, fibrin, and collagen; and Prussian blue stain was used to quantify iron. To assess the reliability of thrombus composition measurements, RBC content was independently requantified using H&E staining in both cerebral venous thrombi and the full set of control thrombi from ischemic stroke patients. The following immunohistochemical stainings were also used: anti-CD61 (for platelets; clone Y2/51; Dako-Agilent), anti-MPO (for neutrophils; polyclonal, ready-to-use; Dako-Agilent), anti-CD3 (for T lymphocytes; polyclonal, ready-to-use; Dako-Agilent), anti-CD20 (for B lymphocytes; clone L26, ready-to-use; Dako-Agilent), anti-CD34 (for endothelial cells; clone OBEnd 10, ready-to-use; Dako-Agilent), anti-CD68 (for macrophages; clone PGM1, ready-to-use; Dako-Agilent), and anti-citH3 (for citrullinated histone H3, a marker of NETs (neutrophil extracellular traps); 1:300, Abcam). All stained sections were digitally scanned using the Panoramic Scan 3DHistec system (Epredia), and stained areas were quantified as a percentage of the positive area relative to the total thrombus area using Orbit Image Analysis Software. The software uses pixel-based classification models that were extensively trained on manually annotated ROIs to reliably distinguish different tissue components. This method was applied uniformly across both conventional histological stains and immunohistochemical analyses. The thrombus area was calculated by delineating a ROI around its edges: the area of the ROI was automatically computed on Orbit, providing an accurate measure of the thrombus size. An experienced pathologist conducted a comprehensive histological assessment of thrombi, evaluating both their maturation stage and the distribution patterns of NETs. Thrombus age was classified into three progressive phases according to the dominant histological features, based on criteria originally developed for deep vein thrombosis (DVT) and adapted for EVT-derived material; as the method was initially designed for autopsy specimens, certain features—such as the endothelial interaction—may be only partially assessable in thrombi retrieved during EVT [15, 16]. In the acute phase (Phase 1), thrombi showed preserved and aggregated RBCs, early leukocyte pyknosis, and initial fibrin layering. The subacute phase (Phase 2) was marked by the appearance of ghost RBCs, hemosiderin-laden macrophages, nuclear debris, and signs of fibrin remodeling often accompanied by measurable collagen deposition. In the chronic stage (Phase 3), thrombi became hyalinized with dense, fiber-rich connective tissue and only sparse residual leukocytes. NETs were morphologically evaluated and classified into three patterns [17] reflecting variations in their extracellular deployment, which likely mirror underlying differences in the dynamics of NET release, potentially shaped by the nature and intensity of the inflammatory trigger [18]. In the cell-like pattern, NETs are localized within and close around the neutrophils. In the filopodia-like pattern, they extend outward in slender, thread-like projections. In the web-like pattern, NETs are diffusely spread, forming widespread extracellular networks.

### 2.4. Statistical Analysis

Categorical variables were described with frequencies and percentages, and continuous variables were described with mean and standard deviation (SD) or median with interquartile range (IQR). We performed univariate comparisons of clinical variables, radiological variables, and thrombus components between venous thrombi and arterial thrombi (CVT vs. LAA and CE). Univariate tests were chosen based on variable type and distribution. Fisher's exact test was used for categorical variables; for continuous variables, the Shapiro–Wilk test determined distribution, guiding the use of either *t*-test or the Mann–Whitney *U* test. Spearman's rank correlation coefficient was used to assess the relationship between mean thrombus density (rHU) and the main thrombus components (RBC, fibrin, collagen, and the RBC-to-fibrin ratio), as well as the concordance of RBC quantification between H&E and Picro Mallory staining, and the intra- and inter-rater reliability of rHU measurements. Statistical analyses were performed using Stata software, Version 18 (StataCorp LLC, College Station, TX, United States). The significance level was set at *p* < 0.05. Biorender was used for generating comparative figures of histological components in venous and arterial thrombi, facilitating data visualization.

### 2.5. Standard Protocol Approvals, Registrations, and Patient Consents

This study received approval from the local ethical committee (Ethics Committee Milano Area 3, Protocol Number: 349-18052022). Patients were duly apprised that all data obtained during routine clinical practice would be utilized for research endeavors and subsequently granted their written informed consent. This study is reported in accordance with the Strengthening the Reporting of Observational Studies in Epidemiology (STROBE) guideline [19].

## 3. Results

Seven patients underwent EVT for CVT during the study period—six at Niguarda Hospital and one at San Raffaele Hospital. Two patients initially treated at Niguarda (but subsequently hospitalized in other institutions) were lost to follow-up and could not be contacted to obtain retrospective consent for study inclusion. Clinical, radiological, and thrombus composition data for the remaining five patients are summarized in Table 1. All cases involved extensive venous system thrombosis, and four out of five patients had received anticoagulant therapy before undergoing EVT. Baseline NIHSS scores ranged from 0 to 5, with four cases showing rHU > 1.00 and grossly visible CT hyperdensity. Three out of five thrombi were classified as recent (Phase 1) and two as subacute (Phase 2); the etiologies of CVT included parainfectious thrombosis, activated protein C resistance, smoking combined with estrogen–progestin therapy, and one cryptogenic case. Endovascular procedures were performed under general anesthesia, primarily using aspiration, achieving complete or near-complete recanalization in all cases. No EVT-related complications were observed, with 90-day mRS scores ranging from 0 to 1, except for one fatality.

**Figure 1:**
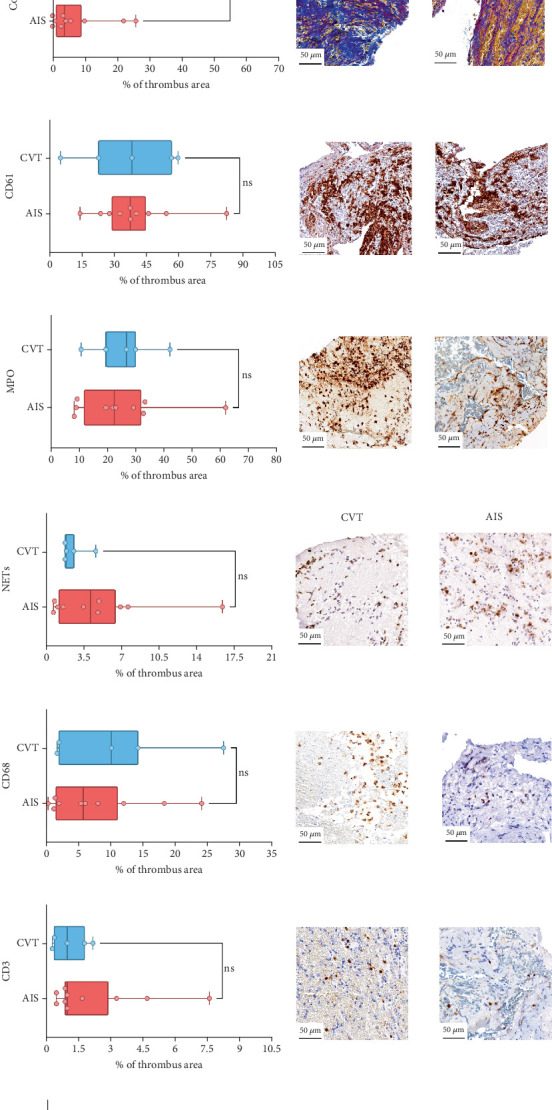
Thrombus assessment and composition of CVT- and AIS-related thrombi. (a) Venous thrombi (CVT) were significantly larger than arterial thrombi (AIS) (*p* = 0.028), as illustrated by both the bar chart and low-magnification H&E-stained sections. Picro Mallory staining shows that (b) red blood cell content (yellow) was comparable between groups (*p* = 0.729), (c) fibrin (pink/purple) was significantly lower in CVT thrombi (*p* = 0.036), and (d) collagen content (blue) did not differ significantly (*p* = 0.129). Immunohistochemistry demonstrated similar levels of (e) platelets (CD61+, *p* = 0.390) and (f) neutrophils (MPO+, *p* = 0.548) in venous and arterial thrombi. (g) NET content (CitH3+) was comparable between groups (*p* = 0.679). No significant differences were observed in (h) macrophage (CD68+, *p* = 0.514), (i) T-cell (CD3+, *p* = 0.440), or (j) B-cell (CD20+, *p* = 0.324) infiltration. (k) Endothelial cells (CD34+) were more abundant in CVT thrombi, though not significantly (*p* = 0.310). (l) Iron deposits, assessed via Prussian blue staining, were minimal and comparable across groups (*p* = 0.953).

Differences in clinical, radiological, and procedural characteristics between AIS and CVT cases are shown in Supporting Information 2: Table S1. Tandem occlusions occurred in four of 10 AIS cases (40%). Intracranial occlusions were located in the dominant M2 in one case (10%), in M1 in four cases (40%), and in the intracranial segment of the internal carotid artery in the remaining 50%. The comparison between the histological composition of CVT and AIS thrombi is illustrated in Figure 1 and also reported in Supporting Information 2: Table S2. Venous thrombi were significantly larger than arterial thrombi, with a median thrombus surface area of 185.6 (IQR 83.0–237.9) versus 21.8 mm^2^ (IQR 8.8–77.8) in arterial thrombi (*p* = 0.028) and had a lower fibrin content (median 16.6% [IQR 13.9–31.5] vs. 46.5% [25.1–49.5], *p* = 0.036). No significant differences were observed in the content of RBCs (Picro Mallory; *p* = 0.729), collagen (Picro Mallory; *p* = 0.129), platelets (CD61; *p* = 0.390), neutrophils (MPO; *p* = 0.548), NETs (CitH3; *p* = 0.679), macrophages (CD68; *p* = 0.514), T lymphocytes (CD3; *p* = 0.440), B lymphocytes (CD20; *p* = 0.324), endothelial cells (CD34; *p* = 0.310), or iron (Prussian blue stain; *p* = 0.953) between venous and arterial thrombi. The morphology of NETs was similar between CVT and AIS cases (*p* = 0.504).

The correlation between thrombus histological components and CT-based relative vascular hypoattenuation (rHU) in CVT cases is reported in Table 2 and graphically reported in Supporting Information 1: Figure S1. A low fibrin content and a high RBC-to-fibrin ratio (correlation coefficients *R* −0.9 and R 0.9, respectively; *p* = 0.047 for both) were significantly correlated with rHU.

A strong positive correlation was observed between H&E- and Picro Mallory–based RBC quantifications (Spearman's *ρ* = 0.85, *p* = 0.03). Additionally, there was strong intrarater (Spearman's *ρ* = 0.95, *p* < 0.001) and inter-rater (Spearman's *ρ* = 0.80, *p* = 0.01) agreement in rHU measurements.

## 4. Discussion

Our study is the first to analyze thrombi histology after EVT in CVT, compare them with AIS cases, and evaluate histology–radiology correlation in CVT. In this exploratory study, CVT thrombi showed heterogeneous histological maturation, ranging from recent to subacute stages, and were rich in RBCs, with significantly less fibrin and larger size compared to arterial thrombi. Fibrin content and the RBC-to-fibrin ratio showed a significant correlation with CT density. Our results provide new insights into the pathophysiology of CVT, elucidating their composition and relationship with CT imaging characteristics.

A key finding of our study is the significant difference in thrombus size between CVT and AIS patients, with venous thrombi being much larger than arterial ones. This is likely due to different pathophysiological processes. In the venous system, slower blood flow and more compliant venous sinuses allow thrombi to grow longer before becoming symptomatic and requiring intervention [20, 21]. In contrast, in the arterial system, where blood flow is faster and vessel walls are less compliant, thrombi are smaller and more compact when symptomatic. This pattern is further supported by the time from symptom onset to EVT in our study, which ranged from 19 to 290 h in CVT cases. While this observation is intuitive and expected given the extent of thrombosis seen in neuroradiological studies before EVT, our study is the first to show a concrete statistically significant size difference between CVT and AIS thrombi, deepening the understanding of how anatomical and hemodynamic factors influence thrombus development. This finding is also consistent with recent autopsy-based evidence demonstrating that thrombus size varies by vascular territory, with thrombi from DVT and pulmonary embolism (PE) generally larger than those from acute myocardial infarction, large-artery atherosclerotic stroke, or CE stroke [22]. Although based on ex vivo material, our results align with this broader pattern and suggest that cerebral venous thrombi may share key morphological characteristics with thrombi formed in other low-flow venous systems. Recognizing these differences may help refine device selection and procedural strategies in EVT for CVT.

In addition, we observed that some venous thrombi showed signs of advanced organization, such as collagen deposition, even when retrieved within relatively short onset-to-EVT intervals. This reflects the overall histological heterogeneity within our sample (which included both recent and subacute CVT thrombi) and suggests that CVT thrombi may begin to develop prior to the appearance of overt clinical symptoms, with symptom onset reflecting a later stage in thrombus progression. A similar phenomenon has been described in DVT, where thrombi often display heterogeneous composition with both recent and organizing components, supporting a staggered maturation process [23]. These observations underscore the importance of integrating histological analysis with clinical timing to better understand the natural history of CVT.

We also observed a notable difference in fibrin content between CVT and AIS thrombi, with venous thrombi having significantly lower fibrin levels. This aligns with prior studies showing that peripheral venous thrombi generally contain less fibrin than arterial thrombi [24]. Indeed, arterial and venous thrombi have distinct compositions: venous thrombi, or “red thrombi,” are rich in RBCs, while arterial thrombi, or “white thrombi,” are richer in fibrin and platelets [25]. The lower fibrin content in CVT thrombi likely reflects the low-shear conditions of venous circulation, favoring RBC accumulation. In contrast, arterial thrombi formed under high-shear conditions are more compact and fibrin-rich. Our study is the first to confirm these differences in CVT thrombi, providing deeper insights into venous thrombosis within the cerebral circulation. Future studies are needed to determine whether the composition of CVT thrombi is comparable to that of DVT. However, such a direct comparison is currently limited by the infrequent use of mechanical thrombus retrieval in the cases of DVT or PE. Numerous studies on thrombus composition in AIS have demonstrated its potential utility in elucidating stroke etiology, predicting the effectiveness of recanalization therapies, and even anticipating clinical outcomes [26–28]. Translating these insights to the context of CVT could offer similarly valuable diagnostic and prognostic information. However, achieving this goal will require larger, multicenter studies with broader patient samples to validate and expand upon our preliminary findings.

In arterial thrombi, CT density has been linked to composition, with hyperdense thrombi typically rich in RBCs and associated with better recanalization outcomes [7]. While a similar association in CVT cases might be expected, no data have existed until now. Our study confirms that hyperdense CVT thrombi on NCCT are also rich in RBCs and low in fibrin. Indeed, we found that relative vascular hypoattenuation was significantly correlated with both a higher RBC-to-fibrin ratio and lower fibrin content. This association likely reflects the differing x-ray attenuation properties of thrombus components; hemoglobin-rich RBCs contribute significantly to CT density, while fibrin has much lower radiopacity. However, no significant correlation was observed between CT attenuation and RBC content alone. This may be explained by previous findings showing that thrombus attenuation on NCCT does not increase linearly with RBC presence, and a density threshold (typically above 50% RBC content) may be required to influence HU values [29]. In small samples, the RBC-to-fibrin ratio may provide a more sensitive metric, balancing the relative contributions of hyper- and hypoattenuating components. In any case, the observed correlation between thrombus composition and CT attenuation helps anticipate thrombus composition based on CT imaging and predicts thrombus behavior during procedures. Identifying thrombus characteristics via imaging could lead to more tailored treatments, guiding device selection for EVT and identifying patients likely to benefit from early intervention.

Our study has several limitations that should be considered when interpreting the findings. The small sample size reflects the rarity of CVT, which occurs in 1–2 cases per 100,000 people annually, with even fewer requiring EVT, as most respond to anticoagulation [2]. Therefore, our sample size should be considered within the context of the rarity of both the condition and the use of EVT. Nonetheless, the significance of this study extends beyond the limited number of patients, as it is the first of its kind and serves as a foundation for future large-scale studies, bolstered by collaborations among multiple centers. Four out of five patients had received anticoagulant therapy prior to EVT, which may have influenced the observed thrombus composition: the decision to proceed with endovascular treatment was guided by the combination of extensive thrombotic involvement and poor response to anticoagulation, despite a relatively mild clinical presentation (NIHSS scores 0–5). This reflects a specific subset of severe, nonresponsive CVT cases, which may influence the interpretation of our findings. The heterogeneity of CVT etiologies (from parainfectious to cryptogenic) further highlights the exploratory nature of the study. Thus, our findings should be seen as hypothesis-generating rather than conclusive, reflecting a narrower subset of the disease. A comparative analysis between thrombus volume estimated from neuroradiological imaging and the ex vivo histological measurements would be of great interest. Unfortunately, we were not able to perform a standardized volumetric assessment on radiological images. Moreover, due to the variable density and often indistinct borders of both venous and arterial thrombi, manual segmentation would lack sufficient precision and reproducibility. Future studies integrating refined imaging techniques and automated segmentation tools may help clarify the relationship between radiological thrombus burden and actual thrombus size. In the meantime, it is worth noting that endovascular procedures achieved complete or near-complete recanalization in all cases, supporting the likelihood that the retrieved thrombotic material closely approximates the full extent of the in situ thrombus. Other limitations of the study include its retrospective design, which introduces potential selection bias. Nonuniform thrombus density, the inability to perform histological analysis at the exact NCCT density sampling point, and preanalytical variations from manual handling may affect result interpretation. Finally, while compositional differences between CVT and AIS thrombi likely reflect their distinct anatomical and hemodynamic environments (venous low-flow vs. arterial high-shear conditions), the longer onset-to-treatment times in CVT may have further influenced thrombus organization. This temporal factor could partially account for some histological differences but should be acknowledged as an inherent limitation when comparing arterial and venous thrombi. Despite all these limitations, our study provides valuable insights by addressing the pathophysiology of this rare condition, which represents a significant knowledge gap [30]. It lays the groundwork for future large-scale research aimed at deepening the understanding of CVT and guiding more targeted treatment strategies.

## 5. Conclusion

Although exploratory in nature, our study is the first to investigate the composition of thrombi obtained from EVT in cases of CVT, compare them with those from AIS cases, and evaluate their histology–radiology correlation. We found that CVT thrombi are larger than AIS thrombi, characterized by a high RBC content and lower fibrin levels, which correspond to their CT density. This study enhances the understanding of CVT pathophysiology and underscores the need for larger studies on this topic.

## Figures and Tables

**Table 1:** Description of clinical, radiological, and histopathological features in five cerebral venous thrombosis cases.

	**Case 1**	**Case 2**	**Case 3**	**Case 4**	**Case 5**
*General characteristics*					
Anticoagulant pre-EVT	Yes	Yes	Yes	No	Yes
Baseline NIHSS score	2	5	0	3	3
CVT site	Right transverse, sigmoid sinuses, and jugular vein	Superior sagittal sinus, torcular, right internal cerebral vein, right and left transverse sinuses, left sigmoid sinus, and straight sinus	Superior sagittal sinus, straight sinus, and transverse sinuses	Sigmoid sinuses, jugular bulbs, and right internal jugular vein	Superior sagittal sinus extending to the left transverse-sigmoid-jugular vein
CT hyperdensity	No	Yes	Yes	Yes	Yes
Mean ipsilateral HU	27	64	63	61	64
Mean controlateral HU	47	46	51	50	55
rHU	0.58	1.40	1.23	1.22	1.16
Etiology	Activated protein C resistance	Smoking habit and estradiol/progesterone-releasing intrauterine device	Parainfectious thrombosis	Parainfectious thrombosis	Cryptogenic
90-day mRS	0	0	0	6	1

*EVT procedural details*					
Onset to EVT (hours)	26	98	290	125	19
First device	Aspiration	Aspiration	Aspiration	Aspiration	Aspiration
Anesthesia	General anesthesia	General anesthesia	General anesthesia	General anesthesia	General anesthesia
Overall technique	Aspiration	Aspiration	Aspiration	Aspiration plus stentriever	Aspiration plus stentriever
Number of passes	1	1	2	> 5	> 5
EVT-related complications	No	No	No	No	No

*Histopathological and immunohistochemical characteristics*					
Thrombus area	186 mm^2^	305 mm^2^	238 mm^2^	82 mm^2^	83 mm^2^
Thrombus age	Phase 2	Phase 1	Phase 2	Phase 1	Phase 1
Red blood cells (%)	37.7	83.9	38.4	75.8	57.5
Fibrin (%)	33.2	13.9	10.7	16.6	31.5
Collagen (%)	29.1	2.1	50.9	7.6	10.9
Anti-CD61 (platelets, %)	56.9	38.5	59.9	5.3	23.0
Anti-MPO (neutrophils, %)	26.8	30.0	42.2	10.6	10.9
Anti-citH3 (NETs) (%)	1.8	1.9	1.8	4.6	2.6
NET pattern	Cell-like	Cell-like	Filopodia-like	Cell-like	Cell-like
Anti-CD68 (macrophages, %)	14.2	2.0	27.5	10.1	1.7
Anti-CD3 (T-cells) (%)	1.8	2.2	0.4	0.3	1.0
Anti-CD20 (B-cells) (%)	0.1	0.7	0.4	0.4	0.2
Anti-CD34 (endothelial cells, %)	2.8	0.0	0.3	2.7	1.8
Iron (%)	1.4	0.0	0.2	0.0	0.0

Abbreviations: CVT, cerebral venous thrombosis; EVT, endovascular treatment; mRS, Modified Rankin Scale; NETs, neutrophil extracellular traps; NIHSS, National Institutes of Health Stroke Scale; rHU, relative Hounsfield unit.

**Table 2:** Correlation between thrombus histological components and CT-based relative vascular hypoattenuation (rHU).

	**Total (** **N** = 15**)**		**CVT (** **N** = 5**)**		**AIS (** **N** = 10**)**	
	**R** ** coefficient**	**p** ** value**	**R** ** coefficient**	**p** ** value**	**R** ** coefficient**	**p** ** value**
RBC-to-fibrin ratio	0.115	0.704	0.900	0.047	−0.048	0.909
Red blood cells	0.313	0.294	0.700	0.190	0.143	0.731
Fibrin	0.126	0.677	−0.900	0.047	0.191	0.645
Collagen	−0.758	0.004	−0.400	0.495	−0.667	0.073

Abbreviations: AIS, acute ischemic stroke; CVT, cerebral venous thrombosis; IQR, interquartile range.

## Data Availability

The data that support the findings of this study are available on request from the corresponding author. The data are not publicly available due to privacy or ethical restrictions.

## Supporting Information

Supporting Information 1 Figure S1: Correlation between rHU and main thrombus components in CVT cases. Figure S1 demonstrates the correlation between CT-based relative Hounsfield units (rHUs) and the main histological components of the thrombi, including red blood cells (RBC), fibrin, and collagen, as well as the RBC-to-fibrin ratio. On the left, an example of a grossly hyperdense thrombus is shown; as indicated by the Picro Mallory staining below, its composition is rich in RBCs. On the right, a macroscopically hypodense thrombus is presented, with its corresponding Picro Mallory staining beneath it, revealing a predominance of fibrin. The red contour in the figure is a graphical aid and does not represent the ROI used to quantify thrombus density on CT.

Supporting Information 2 Table S1: Comparison of clinical, demographic, radiological, and laboratory characteristics between patients with cerebral venous thrombosis (CVT) and acute ischemic stroke (AIS), including EVT-related data and outcomes. Table S2: Comparative analysis of thrombus composition between CVT and AIS patients, including quantitative measures of cellular and molecular components and immunohistochemical markers.

## References

[1] Ferro J. M., Canhao P., Stam J., Bousser M. G., Barinagarrementeria F., Investigators I. (2004). Prognosis of Cerebral Vein and Dural Sinus Thrombosis: Results of the International Study on Cerebral Vein and Dural Sinus Thrombosis (Iscvt). *Stroke*.

[2] Coutinho J. M. (2015). Cerebral Venous Thrombosis. *Journal of Thrombosis and Haemostasis*.

[3] Saposnik G., Barinagarrementeria F., Brown R. D. (2011). Diagnosis and Management of Cerebral Venous Thrombosis: A Statement for Healthcare Professionals From the American Heart Association/American Stroke Association. *Stroke*.

[4] Coutinho J. M., Zuurbier S. M., Bousser M. G. (2020). Effect of Endovascular Treatment With Medical Management vs Standard Care on Severe Cerebral Venous Thrombosis: The to-Act Randomized Clinical Trial. *JAMA Neurology*.

[5] Siddiqui F. M., Dandapat S., Banerjee C. (2015). Mechanical Thrombectomy in Cerebral Venous Thrombosis: Systematic Review of 185 Cases. *Stroke*.

[6] Bacigaluppi M., Semerano A., Gullotta G. S., Strambo D. (2019). Insights From Thrombi Retrieved in Stroke due to Large Vessel Occlusion. *Journal of Cerebral Blood Flow and Metabolism*.

[7] Brinjikji W., Duffy S., Burrows A. (2017). Correlation of Imaging and Histopathology of Thrombi in Acute Ischemic Stroke With Etiology and Outcome: A Systematic Review. *Journal of NeuroInterventional Surgery*.

[8] Fereidoonnezhad B., Dwivedi A., Johnson S., McCarthy R., McGarry P. (2021). Blood Clot Fracture Properties Are Dependent on Red Blood Cell and Fibrin Content. *Acta Biomaterialia*.

[9] Schwarz G., Rizzo A. C., Di Como M. (2024). Exploring Thrombus Composition in Cerebral Venous Thrombosis: The First Case Report With Initial Insights and Implications for Treatment Advancements. *Neurological Sciences*.

[10] Ferro J. M., Bousser M. G., Canhao P. (2017). European Stroke Organization Guideline for the Diagnosis and Treatment of Cerebral Venous Thrombosis-Endorsed by the European Academy of Neurology. *European Stroke Journal*.

[11] Adams H. P., Bendixen B. H., Kappelle L. J. (1993). Classification of Subtype of Acute Ischemic Stroke. Definitions for Use in a Multicenter Clinical Trial. Toast. Trial of Org 10172 in Acute Stroke Treatment. *Stroke*.

[12] Almekhlafi M. A., Mishra S., Desai J. A. (2014). Not All "Successful" Angiographic Reperfusion Patients Are an Equal Validation of a Modified Tici Scoring System. *Interventional Neuroradiology*.

[13] Hacke W., Kaste M., Fieschi C. (1998). Randomised Double-Blind Placebo-Controlled Trial of Thrombolytic Therapy With Intravenous Alteplase in Acute Ischaemic Stroke (Ecass II). Second European-Australasian Acute Stroke Study Investigators. *Lancet*.

[14] van Swieten J. C., Koudstaal P. J., Visser M. C., Schouten H. J., van Gijn J. (1988). Interobserver Agreement for the Assessment of Handicap in Stroke Patients. *Stroke*.

[15] Fineschi V., Turillazzi E., Neri M., Pomara C., Riezzo I. (2009). Histological Age Determination of Venous Thrombosis: A Neglected Forensic Task in Fatal Pulmonary Thrombo-Embolism. *Forensic Science International*.

[16] Semerano A., Dell’Acqua B., Genchi A. (2025). Cerebral Thrombus Analysis as a Useful Diagnostic Tool for Infective Endocarditis in Ischemic Stroke Patients. *European Stroke Journal*.

[17] Heo J. H., Nam H. S., Kim Y. D. (2020). Pathophysiologic and Therapeutic Perspectives Based on Thrombus Histology in Stroke. *Journal of Stroke*.

[18] Zhu S., Yu Y., Ren Y. (2021). The Emerging Roles of Neutrophil Extracellular Traps in Wound Healing. *Cell Death & Disease*.

[19] von Elm E., Altman D. G., Egger M. (2007). The Strengthening the Reporting of Observational Studies in Epidemiology (Strobe) Statement: Guidelines for Reporting Observational Studies. *Lancet*.

[20] Chernysh I. N., Nagaswami C., Kosolapova S. (2020). The Distinctive Structure and Composition of Arterial and Venous Thrombi and Pulmonary Emboli. *Scientific Reports*.

[21] Wilson M. H. (2016). Monro-Kellie 2.0: The Dynamic Vascular and Venous Pathophysiological Components of Intracranial Pressure. *Journal of Cerebral Blood Flow and Metabolism*.

[22] Yamashita A., Gi T., Sato Y. (2025). Histological Differences Among Thrombi in Thrombotic Diseases. *Current Opinion in Hematology*.

[23] Oguri N., Gi T., Nakamura E. (2024). Expression of Fibroblast Activation Protein-*α* in Human Deep Vein Thrombosis. *Thrombosis Research*.

[24] Lippi G., Favaloro E. J. (2018). Venous and Arterial Thromboses: Two Sides of the Same Coin?. *Seminars in Thrombosis and Hemostasis*.

[25] Wolberg A. S., Aleman M. M., Leiderman K., Machlus K. R. (2012). Procoagulant Activity in Hemostasis and Thrombosis: Virchow's Triad Revisited. *Anesthesia and Analgesia*.

[26] Boeckh-Behrens T., Kleine J. F., Zimmer C. (2016). Thrombus Histology Suggests Cardioembolic Cause in Cryptogenic Stroke. *Stroke*.

[27] Sporns P. B., Hanning U., Schwindt W. (2017). Ischemic Stroke: What Does the Histological Composition Tell Us About the Origin of the Thrombus?. *Stroke*.

[28] Shimizu H., Hatakeyama K., Saito K. (2022). Age and Composition of the Thrombus Retrieved by Mechanical Thrombectomy From Patients With Acute Ischemic Stroke Are Associated With Revascularization and Clinical Outcomes. *Thrombosis Research*.

[29] Ding Y. H., Abbasi M., Michalak G. (2021). Characterization of Thrombus Composition With Multimodality Ct-Based Imaging: An In-Vitro Study. *Journal of NeuroInterventional Surgery*.

[30] Coutinho J. M., van de Munckhof A., Aguiar de Sousa D. (2024). Reducing the Global Burden of Cerebral Venous Thrombosis: An International Research Agenda. *International Journal of Stroke*.

