# Supplementary figures and images for: Thrombus Composition in Cerebral Venous Thrombosis
Source: Stroke Res Treat. 2025 Jun 23;2025:8650226. doi: 10.1155/srat/8650226 (PMC12208753; doi:10.1155/srat/8650226)

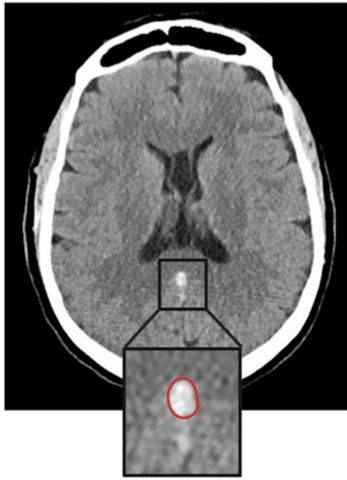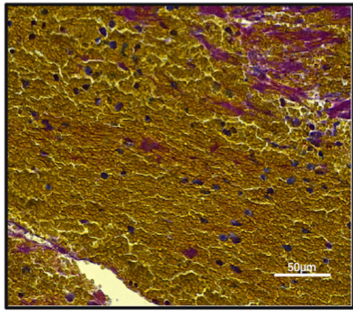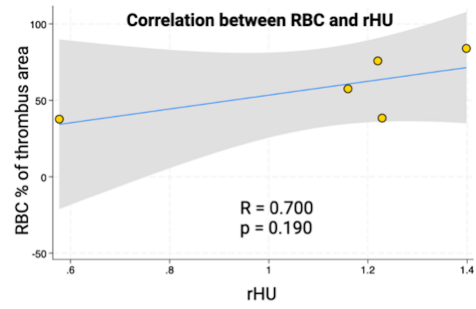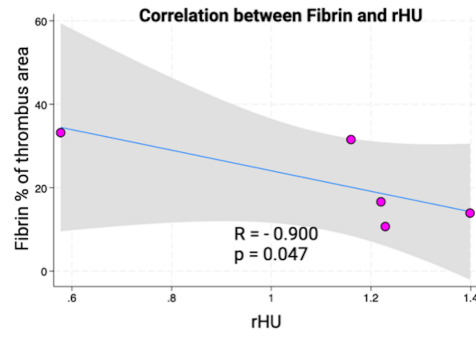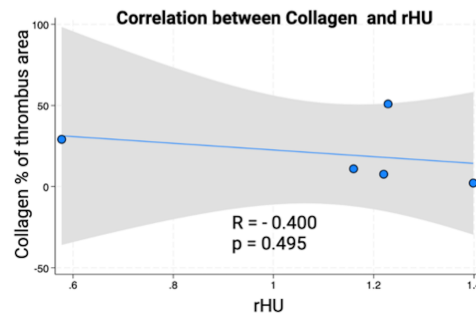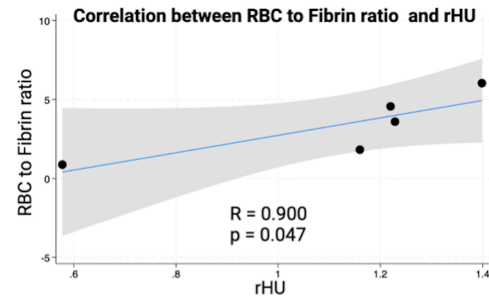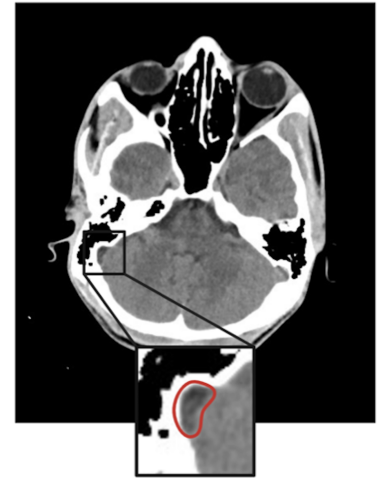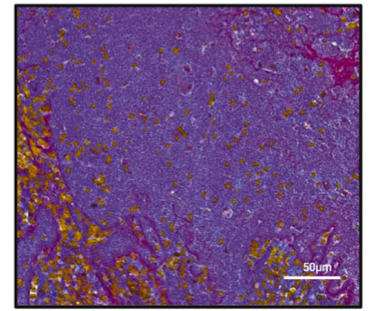

Supplement: Supporting Information 1 — Figure S1: Correlation between rHU and main thrombus components in CVT cases. Figure S1 demonstrates the correlation between CT-based relative Hounsfield units (rHUs) and the main histological components of the thrombi, including red blood cells (RBC), fibrin, and collagen, as well as the RBC-to-fibrin ratio. On the left, an example of a grossly hyperdense thrombus is shown; as indicated by the Picro Mallory staining below, its composition is rich in RBCs. On the right, a macroscopically hypodense thrombus is presented, with its corresponding Picro Mallory staining beneath it, revealing a predominance of fibrin. The red contour in the figure is a graphical aid and does not represent the ROI used to quantify thrombus density on CT. [file 8650226.f1.pdf]
