# Supplementary material for: Thrombus Composition in Cerebral Venous Thrombosis
Source: Stroke Res Treat. 2025 Jun 23;2025:8650226. doi: 10.1155/srat/8650226 (PMC12208753; doi:10.1155/srat/8650226)
Supplement: Supporting Information 2 — Table S1: Comparison of clinical, demographic, radiological, and laboratory characteristics between patients with cerebral venous thrombosis (CVT) and acute ischemic stroke (AIS), including EVT-related data and outcomes. Table S2: Comparative analysis of thrombus composition between CVT and AIS patients, including quantitative measures of cellular and molecular components and immunohistochemical markers. [file 8650226.f2.docx]

*Supplementary Tables to:*

**Thrombus Composition in Cerebral Venous Thrombosis**

Ghil Schwarz MD^1^, Angelo Cascio Rizzo MD^1^, Martina Di Como MS^2^, Amedeo Cervo MD^3^, Antonio Macera MD^3^, Guglielmo Carlo Pero MD^3^, Maria Costanza Aquilano MD^2^, Beatrice dell’Acqua MD^4^, Marco Bacigaluppi PhD^4^, Arturo Chieregato MD^5^, Francesco Ruggieri MD^5^, Emanuela Bonoldi MD^2^, Mariangela Piano MD^3^, Maria Sessa MD^1^, Elio Clemente Agostoni MD^1^

^1^ Department of Neurology and Stroke Unit - ASST Grande Ospedale Metropolitano Niguarda, Milan, Italy

^2^ Department of Hematology, Oncology and Molecular Medicine, ASST Grande Ospedale Metropolitano Niguarda, Milan, Italy

^3^ Department of Neuroradiology – ASST Grande Ospedale Metropolitano Niguarda, Milan, Italy

^4^ Neuroimmunology Unit, Institute of Experimental Neurology, San Raffaele Hospital, Milan, Italy

^5^ Neurointensive Care Unit, ASST Grande Ospedale Metropolitano Niguarda, Milan, Italy

| **Table S1. Comparison of Clinical, Demographic, Radiological, and Laboratory Data in Cerebral Venous Thrombosis (CVT) and Acute Ischemic Stroke (AIS) Patients** | | | | |
| --- | --- | --- | --- | --- |
|  | **Total** | **CVT** | **AIS** | **p value** |
|  | **N=15** | **N=5** | **N=10** | CVT vs AIS |
| **Clinical, Demographics, Radiological and Laboratoristic data** | | | | |
| Age (mean, SD) | 64 (24) [15] | 38 (22) [5] | 77 (10) [10] | **<0.001** |
| Sex (female) | 6/15 (40.0) | 2/5 (40.0) | 4/10 (40.0) | 0.520 |
| Hypertension | 8/15 (53.3) | 1/5 (20.0) | 7/10 (70.0) | 0.100 |
| Diabetes | 2/15 (13.3) | 1/5 (20.0) | 1/10 (10.0) | 0.571 |
| Dyslipidemia | 3/15 (20.0) | 0/5 (0.0) | 3/10 (30.0) | 0.264 |
| Smoking (current) | 3/15 (20.0) | 1/5 (20.0) | 2/10 (20.0) | 0.736 |
| Atrial fibrillation | 4/15 (26.7) | 0/0 (0.0) | 4/10 (40.0) | 0.154 |
| BMI (median [IQR]) | 25.5 (23.9-27.2) [15] | 26.6 (25.5-27.2) [5] | 24.9 (23.9-26.8) [10] | 0.439 |
| Baseline NIHSS (median [IQR]) | 6 (3-15) [15] | 3 (2-3) [5] | 10 (6-17) [10] | **0.008** |
| Hyperdense vascular sign | 11/13 (84.6) | 4/5 (80.0) | 7/8 (87.5) | 0.641 |
| rHU (median [IQR]) | 1.3 (1.2-1.4) [13] | 1.2 (1.2-1.2) [5] | 1.4 (1.2-1.6) [8] | 0.079 |
| IVT (median [IQR]) | 1/15 (6.7) | - | 1/10 (10.0) | - |
| *OTI (median [IQR]; minutes) | 390 (180-1440) [15] | 5760 (1440-7300) [5] | 220 (130-390) [10] | **0.007** |
| OTN (median [IQR] ; minutes) | 260 (260-260) [1] | - | 260 (260-260) [1] | - |
| OTG (median [IQR]; *minutes*) | 480 (300-1560) [15] | 5850 (1560-7500) [5] | 312 (290-480) [10] | **0.007** |
| OTG (median [IQR]; *hours*) | 8 (5 - 26) | 98 (26 - 125) | 5 (5 - 8) |  |
| OTR (median [IQR] ; minutes) | 510 (330-1590) [15] | 5970 (1590-7600) [5] | 385 (320-510) [10] | **0.007** |
| **EVT-related details** |  |  |  |  |
| First endovascular device:  Aspiration  Combined  Stentriever | 7/15 (46.7)  7/15 (46.7)  1/15 (6.7) | 5/5 (100.0)  0/5 (0.0)  0/5 (0.0) | 2/10 (20.0)  7/10 (70.0)  1/10 (10.0) | **0.014** |
| Effective device  Aspiration  Combined  Stentriever | 5/14 (35.7)  7/14 (50.0)  2/14 (14.3) | 4/5 (80.0)  0/5 (0.0)  1/5 (20.0) | 1/9 (11.1)  7/9 (77.8)  1/9 (11.1) | **0.010** |
| Number of passes (median [IQR]) | 1 (1-3) [15] | 2 (1-6) [5] | 1 (1-3) [10] | 0.930 |
| EVT complications | 1/15 (6.7) | 0/5 (0.0) | 1/10 (10.0) | 0.667 |
| General anesthesia | 6/15 (40.0) | 5/5 (100.0) | 1/10 (10.0) | **0.002** |
| **Clinical/Radiological Outcomes** |  |  |  |  |
| 24h-NIHSS (median [IQR]) | 4 (0-7) [15] | 1 (0-3) [5] | 6 (2-12) [10] | 0.187 |
| Symptomatic haemorrhagic transformation | 0/15 (0.0) | 0/5 (0.0) | 0/10 (0.0) | 1.000 |
| 90-day mRS (median [IQR]) | 1 (0-3) [15] | 0 (0-1) [5] | 2 (1-3) [10] | 0.197 |
| *Refers to the pre-EVT imaging, which is the one used for CT density measurements of the thrombus, although it may not necessarily be the first imaging study performed in CVT cases.  AIS, Acute Ischemic Stroke; ASPECTs, Alberta Stroke Program Early CT Score; BMI, Body Mass Index; CVT, Cerebral Venous Thrombosis; EVT, Endovascular Treatment; IQR, Interquartile Range; IVT, Intravenous Thrombolysis; mRS, Modified Rankin Scale; mTICI, Modified Thrombolysis in Cerebral Infarction; NIHSS, National Institutes of Health Stroke Scale; OTG, Onset-to-Groin Puncture Time; OTI, Onset-to-Imaging Time; OTN, Onset-to-Needle Time; OTR, Onset-to-Reperfusion Time; rHU, Relative Hounsfield Units; SD, Standard Deviation. | | | | |

| **Table S2. Comparative Analysis of Thrombus Composition in Cerebral Venous Thrombosis (CVT) and Acute Ischemic Stroke (AIS)** | | | | |
| --- | --- | --- | --- | --- |
|  | **Total** | **CVT** | **AIS** | **p value** |
|  | **N=15** | **N=5** | **N=10** | CVT vs AIS |
| **Thrombus composition** |  |  |  |  |
| Thrombus area (median [IQR]) | 77 (18-185) [15] | 185.6 (83.0-237.9) [5] | 21.8 (8.8-77.8) [10] | **0.028** |
| Red blood cell / Fibrin ratio (median [IQR]) | 1.8 (0.9-6.0) [15] | 3.6 (1.8-4.6) [5] | 1.1 (0.94-6.5) [10] | 0.679 |
| Red blood cells (median [IQR]) | 50.5 (38.4-70.2) [15] | 57.5 (38.4-75.8) [5] | 50.5 (43.8-70.1) [10] | 0.729 |
| Fibrin (median [IQR]) | 31.5 (14.1-48.9) [15] | 16.6 (13.9-31.5) [5] | 46.5 (25.1-49.5) [10] | **0.036** |
| Collagen (median [IQR]) | 5.6 (2.1-21.9) [15] | 10.9 (7.6-29.1) [5] | 3.7 (0.7-9.9) [10] | 0.129 |
| Anti-CD61 (Platelets) (median [IQR]) | 37.8 (23.9-54.8) [15] | 38.5 (23.0-56.9) [5] | 37.7 (28.0-46.2) [10] | 0.390 |
| Anti-MPO (Neutrophils) (median [IQR]) | 23.0 (10.9-32.8) [15] | 26.8 (19.6-30.0) [5] | 22.5 (9.5-32.8) [10] | 0.548 |
| Anti-citH3 (NETs) (median [IQR]) | 2.6 (1.6-4.9) [15] | 1.9 (1.8-2.6) [5] | 4.1 (1.1-6.7) [10] | 0.679 |
| Anti-CD68 (Macrophages) (median [IQR]) | 6.1 (1.7-14.2) [15] | 10.1 (2.0-14.2) [5] | 5.7 (1.4-12.0) [10] | 0.514 |
| Anti-CD3 (T-cells) (median [IQR]) | 1.0 (0.5-2.2) [15] | 1.0 (0.4-1.8) [5] | 1.0 (0.9-3.3) [10] | 0.440 |
| Anti-CD20 (B-cells) (median [IQR]) | 0.4 (1.8-0.6) [15] | 0.4 (0.2-0.4) [5] | 0.5 (0.2-0.6) [10] | 0.324 |
| Anti-CD34 (Endothelial cells) (median [IQR]) | 0.5 (0.3-1.3) [15] | 0.5 (0.3-1.8) [5] | 0.6 (0.3-1.1) [10] | 0.679 |
| Iron (median [IQR]) | 0.0 (0.0-0.1) [15] | 0.0 (0.0-0.2) [5] | 0.0 (0.0-0.1) [10] | 0.953 |
| NETs pattern  Cell-like  Filopodia-like  Web-like | 9/15 (60.0)  5/15 (33.3)  1/15 (6.7) | 4/5 (80.0)  1/5 (20.0)  0/5 (0.0) | 5/10 (50.0)  4/10 (40.0)  1/10 (10.0) | 0.504 |
| AIS: Acute Ischemic Stroke; CVT: Cerebral Venous Thrombosis; IQR: Interquartile Range; NETs: Neutrophil Extracellular Traps; rHU: Relative Hounsfield Units; citH3: Citrullinated Histone H3. | | | | |
